# *Streptococcus suis* serotype 2 enolase interaction with host brain microvascular endothelial cells and RPSA-induced apoptosis lead to loss of BBB integrity

**DOI:** 10.1186/s13567-020-00887-6

**Published:** 2021-02-22

**Authors:** Hongtao Liu, Siyu Lei, Li Jia, Xiaojing Xia, Yingying Sun, Hexiang Jiang, Rining Zhu, Shuguang Li, Guanggang Qu, Jingmin Gu, Changjiang Sun, Xin Feng, Wenyu Han, Paul R. Langford, Liancheng Lei

**Affiliations:** 1 Key Laboratory of Zoonosis, Ministry of Education, Institute of Zoonosis/College of Veterinary Medicine, Jilin University, Changchun, Jilin 130062 People’s Republic of China; 2 School of Basic Medicine, Jilin University, Changchun, 130021 China; 3 Shandong Binzhou Animal Science and Veterinary Medicine Academy, Binzhou, Shandong 256600 People’s Republic of China; 4 Section of Paediatric Infectious Disease, Imperial College London, London, W2 1PG UK; 5 College of Animal Science, Yangtze University, Jingzhou, Hubei 434023 People’s Republic of China

**Keywords:** *Streptococcus suis* serotype 2, Enolase, Apoptosis, RPSA, Blood brain barrier, Meningitis

## Abstract

Host proteins interacting with pathogens are receiving more attention as potential therapeutic targets in molecular medicine. *Streptococcus suis* serotype 2 (SS2) is an important cause of meningitis in both humans and pigs worldwide. SS2 Enolase (Eno) has previously been identified as a virulence factor with a role in altering blood brain barrier (BBB) integrity, but the host cell membrane receptor of Eno and The mechanism(s) involved are unclear. This study identified that SS2 Eno binds to 40S ribosomal protein SA (RPSA) on the surface of porcine brain microvascular endothelial cells leading to activation of intracellular p38/ERK-eIF4E signalling, which promotes intracellular expression of HSPD1 (heat-shock protein family D member 1), and initiation of host-cell apoptosis, and increased BBB permeability facilitating bacterial invasion. This study reveals novel functions for the host-interactional molecules RPSA and HSPD1 in BBB integrity, and provides insight for new therapeutic strategies in meningitis.

## Introduction

*Streptococcus suis* (SS) is a newly emerging zoonotic pathogen that can cause meningitis, endangering health in both humans and pigs. Approximately 1600 cases of SS have been reported in 30 countries, including Western Europe, Canada, and the United States [1]. The number of people infected in China has reached 300 [2]. SS serotype 2 (SS2), the most virulent serotype, is the most commonly isolated in human infection cases, accounting for 74.7% (*n* = 1642) [3, 4]. Some countries have a higher isolation rate of SS2, such as Thailand, where 94.6% has been reported [5].

SS2 can pass through the blood brain barrier (BBB) formed by brain microvascular endothelial cells (BMEC) and/or choroid plexus epithelial cells. In the case of BMECs, SS2 interaction induces serine/threonine kinase activity that affects the expression of E3 ubiquitin ligase HECTD1, which subsequently increases the degradation of claudin-5, thus enabling SS2 to traverse the BBB [6]. Once bacteria enter the brain tissue, meningitis is the most serious clinical manifestation of SS2 infection; which is difficult to treat because of the difficulty of delivery of therapeutic drugs to the brain, but also the associated long term sequelse [7]. The SS virulence factor suilysin may also have a role in inducing BMEC injury, and passage across the BBB to the central nervous system (CNS) [8, 9]. Understanding the host and bacterial interaction that underpins the traversal of SS2 across the BBB, is important for formulating new prevention and treatment measures.

Enolase (Eno) is a ubiquitous protein in living organisms, including eukaryotic and prokaryotic cells. In bacteria, Eno is normally considered to be a cytoplasmic catalytic enzyme in the bacterial glycolysis pathway. SS Eno was initially identified by a proteomics approach [10], subsequently shown to be an octamer [11], and found in the cytoplasm, in culture supernatants, and on the bacterial surface. Eno is considered a virulence factor because it contributes to bacterial adherence, colonisation of mucosal surfaces, and invasion of host cells [12, 13]. Eno can also induce an immune response that provides protection against SS challenge [12, 14]. Furthermore, in vitro, SS Eno binds fibronectin and plasminogen in a lysine-dependent manner, and anti-Eno antibody prevents SS from adhering to and invading porcine brain microvascular endothelial cells (PBMECs), the major constituent of the pig BBB [15]. Our group demonstrated that Eno binds to an in vitro BBB model comprising co-culture of primary PBMECs and astrocytes [16], and indirectly enhances the permeability of the BBB in vitro and in vivo by stimulating IL-8 secretion [17]. These studies suggest that Eno is involved in the adherence of SS to PBMECs and alters BBB permeability, although the mechanisms were not determined.

40S Ribosome protein SA (RPSA) is found in many kinds of cells with a wide range of cellular locations [18]. It has been reported that RPSA is an important receptor involved in the internalisation of some viruses into host cells, such as Dengue virus [19]. RPSA is enriched dramatically in brain tissue after SS2 infection [20], suggesting it has a role in bacterial meningitis.

The human brain microvascular endothelial cell line hCMEC/D3 is also a model of the human BBB that can be easily grown in vitro, is amenable to cellular and molecular studies on pathological and drug transport mechanisms that are relevant to the CNS [21], and widely used to study SS2-induced meningitis [22, 23]. PBMECs can be successfully separated and used to study the mechanism of SS2 breaking through BBB [16]. Thus both porcine and human brain microvascular endothelial cells are suitable to study SS2 interaction with the BBB [16, 22, 23], and in this study we have determined the effect of SS2 protein Eno on both porcine (PBMECs) and subsequently human (hCMEC/D3) cells.

In this study, the interaction between SS2 Eno and PBMECs was analysed, and RPSA was identified as an Eno-binding protein. It was found that the binding of RPSA with Eno promotes phosphorylation of p38 and ERK mitogen-activated protein kinase (MAPK), and consequently promotes phosphorylation of eukaryotic initiation factor 4E (eIF4E), which leads to increased expression of HSPD1, and activatation of caspase-8 followed by caspase-3. This process induces PBMECs to be apoptotic, resulting in the impairment and increased permeability of the BBB, and the promotion of bacterial penetration through the barrier. This study provides a greater understanding of the novel functions of the host-interactional molecule RPSA and the pathogenic role of bacterial Eno in meningitis, and it forms the basis for new therapeutic strategies to treat meningitis.

## Materials and methods

### Bacterial strains and culture conditions

SS2 strains JZLQ022 (our collection) and CVCC606 (purchased from the China Veterinary Culture Collection Center [24]) were isolated from brain material from porcine meningitis cases. Strains were grown on tryptic soy agar (TSA) plates with 5% newborn bovine serum for 10 h at 37 °C. Single colonies were transferred into 3 mL of tryptic soy broth (TSB) with 5% newborn bovine serum, and incubated for 8 h at 37 °C with agitation. All* Escherichia coli* (*E. coli*) strains were grown on Luria–Bertani (LB) Broth agar plates and incubated for 10 h at 37 °C. Single colonies were transferred into 5 mL of LB broth and incubated for 8 h at 37 °C with agitation.

### Effects of Eno, SS2, and BL21-Eno on the integrity of the BBB co-culture model

PBMECs and astrocyte cells (ACs) were obtained and used for establishment of a porcine BBB model as previously described [16]. Eno, anti-Eno (Rabbit, IgG), or anti-Neg (rabbit, isotype IgG) (0.5 μM) was added to the upper chamber of the BBB model (*n* = 3), and 10^5^ CFU of SS2 CVCC606 was added after 0.5 h. SS2 in the lower chamber of the model was counted after 3 h. When investigated, 100 μL of DMEM with 10^5^ CFU of BL21-pET23a::Eno (BL21 with pET23a vector as control group) was added to the cell Petri dish after 0.5 h.

### Co-immunoprecipitation (Co-IP) of Eno and RPSA

Human embryonic kidney: 293T cells were transfected with pEGFP-C::Eno and pCMV-3Flag-9::RPSA for 24 h, and lysed with NP-40 lysis buffer on ice. The lysate was transferred into a 1.5-mL Eppendorf tube and centrifuged at 12 000 × *g* for 10 min at 4 °C to collect the supernatant. The total proteins were incubated with anti-FLAG^®^ M2 Magnetic beads (Sigma) overnight at 4 °C, washed three times in NP-40 lysis buffer, and resuspended in 50 μL 1 × SDS loading buffer. Eno and RPSA expression were identified by Western blotting.

### Identification of PBMEC proteins interacting with Eno

Based on the instructions of the Pierce™ Crosslink Magnetic IP/Co-IP Kit (Thermo, 88805), mouse anti-His IgG was incubated with beads at 37 °C for 2 h, and un-crosslinked anti-His was removed using lysis/wash buffer (provided in the kit). His-Eno (His tag was used for the control group) was incubated with beads/anti-His, and un-crosslinked His-Eno or His tag was washed with lysis/wash buffer. A 1 × SDS loading buffer was added to the pellets of anti-His/His-Eno or His tag beads, and then they were boiled at 100 °C for 5 min.

### Immunofluorescence of PBMECs to detect Eno-RPSA interaction

PBMECs: were treated with recombinant Eno with 6 × N-terminal His tags for 30 min, fixed at room temperature for 30 min with 4% paraformaldehyde, washed in PBS three times, incubated in 0.02% Triton X-100 for 20 min, washed in PBS three times, stabilised in 10% goat serum at room temperature for 30 min, washed in PBS three times, incubated with 1:100 mouse anti-His IgG and rabbit anti-RPSA at 37 °C for 1 h, washed with PBS three times, incubated with 1:1000 Alexa Fluor 488-conjugated goat anti-rabbit and Alexa Fluor 594-conjugated goat anti-mouse IgG at room temperature for 1 h, and then washed with PBS three times. Finally, the cells were stained with 1:10 000 Hoechst for 5 min, washed with PBS three times, and observed by confocal microscopy.

### Immunoelectron microscopic analysis

Cells were fixed in solution with 0.5% glutaraldehyde/2% paraformaldehyde (pH 7.4) for 12 h at 4 °C, washed in PBS three times, and transferred to 1% osmium tetroxide acid for 1 h at 4 °C. Subsequently, the cells were dehydrated with a graded ethanol series (30%, 50%, 70%, 80%, 90%, and 100%) and acetone and then infiltrated with epoxy resin. Epoxy-treated samples were embedded in gelatin capsules and polymerised for 48 h at 60 °C. Following polymerisation, ultrathin Sects. (70 nm thickness) were cut and loaded onto a 300-mesh nickel grid without coating. Prior to incubation with antibodies, the dried ultrathin sections were blocked for 20 min at 4 °C with 1% BSA in PBS. Samples were incubated with rabbit anti-RPSA (1:20 dilution, Proteintech, 14533-1-AP) and anti-Eno (rabbit anti-Eno IgG prepared in the lab) for 120 min. Grids were incubated with colloidal gold-conjugated goat anti-rabbit or mouse IgG (1:40 dilution, source catalogue number) secondary antibody for 120 min at room temperature. All antibodies were diluted with PBS buffer (pH 7.4) containing 1% BSA. After incubation with the primary and secondary antibodies, the sections were contrast stained with 2% aqueous uranyl acetate for 15 min.

### Detection of bacterial loads in brain tissue and survival rate of SS2-infected mice

Healthy 4-to-6-week-old ICR mice (20–22 g) were purchased from Changchun Yisi Experimental Animal Co., Ltd. and randomly divided into groups (*n* = 5 for each group). For the SS2/Anti-Eno group, mice were injected with rabbit anti-Eno IgG (100 μL with 1 μg IgG) intravenously and, 0.5 h later, infected with 1 × 10^7^ CFUs SS2 intravenously. For the SS2/Anti-Negative group, mice were intravenously injected with control rabbit IgG and then infected with 1 × 10^7^ CFU SS2 intravenously 0.5 h later. For the BL21-Eno/Anti-Eno groups, mice were intravenously injected with rabbit anti-Eno IgG and, 0.5 h later, infected with 1 × 10^9^ CFU BL21-pET23a::Eno intravenously. For the BL21-Eno/anti-Negative group, mice were intravenously injected with control rabbit IgG and then infected with 1 × 10^9^ CFU BL21-pET23a intravenously 0.5 h later. Blood and brain tissues were collected from each group 24 h after infection. The blood was anticoagulated with heparin sodium, diluted 10 times, and plated for CFU determination. Aliquots (1 g) of brain tissue were added to 500 μL of PBS and homogenised, and the homogenates were serially diluted tenfold. CFUs were determined after overnight incubation at 37 °C, and survival rate was detected using n = 10.

### SS2 and *E. coli* adhesion and invasion assays

SS2 or BL21-pET23a::Eno were cultured to logarithmic phase, and 1 × 10^5^ CFUs added PBMECs in 12-well plates, followed by incubation at 37 °C for 1 h. The cells were washed with PBS buffer, lysed with 0.02% saponin, and serial dilutions were plated to determine the CFU of adherent bacteria. For invasion, SS2 or BL21-pET23a/pET23a::Eno, PBMECs were washed three times in PBS and then incubated for 30 min in 200 µg/mL ampicillin or 100 µg/mL kanamycin, respectively. After three further washes in PBS and lysis, serial dilutions were plated for CFU determination. All adherence and invasion experiments were performed in triplicate.

### RPSA and/or HSPD1 knockdown using RNAi

Cell-level RNAi assay: PBMECs were transfected with RPSA or HSPD1 siRNA (Additional file 1: Table S1) using the X-gene RNA transfection reagent (Roche, China, 04476093001), and the cell pellet was lysed by NP-40 lysis buffer at 24 h, 48 h, and 72 h after transfection. The cell lysate was lysed using NP-40 lysis buffer and collected by centrifugation at 1200 x *g* (sign x) for 10 min. The expression of the RPSA and HSPD1 proteins was analysed by Western blotting to determine whether the target protein was successfully knocked down. RNAi assay in mouse brains: Fluorescent-labelled AAV2/9 virus carrying the target shRNA (RPSA and/or HSPD1) and control shRNA was synthesised by Shanghai Genechem Co. Ltd and microinjected into the third ventricle of the mice. The mice were continuously monitored after injection with the virus. After 60 days, mouse brain tissue was collected, and frozen sections were observed to determine whether the target protein was knocked down in the brain.

### The establishment of a porcine model of SS2 infection

Eighteen 26-day-old healthy inbred Bama miniature pigs were randomly divided into two groups (*n* = 6): SS2 infection group (200 μL PBS with 5 × 10^6^ CFU JZLQ022) and healthy group (200 μL PBS) by ear intravenous injection [25]. Clinical symptoms including appetite, mental status and body temperature were continuously monitored post infection. The pigs were euthanized with sodium pentobarbital (400 mg/mL at a dose of 500 μL/kg) by ear intravenous injection at the end of the experiment or for humane reasons. The blood, brain tissues and cerebrospinal fluid were collected at post-Mortimer.

### HE staining and immunohistochemical analysis of brain tissues

Brain tissues from mouse and porcine models [25] were fixed in 4% (w/v) polyoxymethylene solution for 48 h prior to histopathological examination. The brains were fixed by perfusion, embedded in paraffin, and stained with HE, acid vanadium fuchsin, cresyl violet, and GFAP [26]. Immunohistochemical analysis was performed with Histostain™-SP Kits (ZSbio, Beijing, China, SPN-9001) according to the manufacturer’s instructions. The primary antibodies used in this assay were rabbit anti-RPSA and -HSPD1 (1:20 dilution, Proteintech), and along rabbit anti-cleaved caspase-3, -phosphorylated p38, -phosphorylated ERK, and -phosphorylated eIF4E. (1:20 dilution, Affinity).

### Statistical data analysis

Statistical analyses were performed using the data analysis function of the Graphpad5 software. One-way ANOVA (*p < 0.05, was used to compare control and test groups with *p* < 0.05 considered statistically significant. Multiple comparisons were performed using the Tukey one-way ANOVA test with *p* < 0.05 being considered significant.

## Results

### Eno destroys BBB integrity and mediates SS2 invasion of the brain

**Figure 1:**
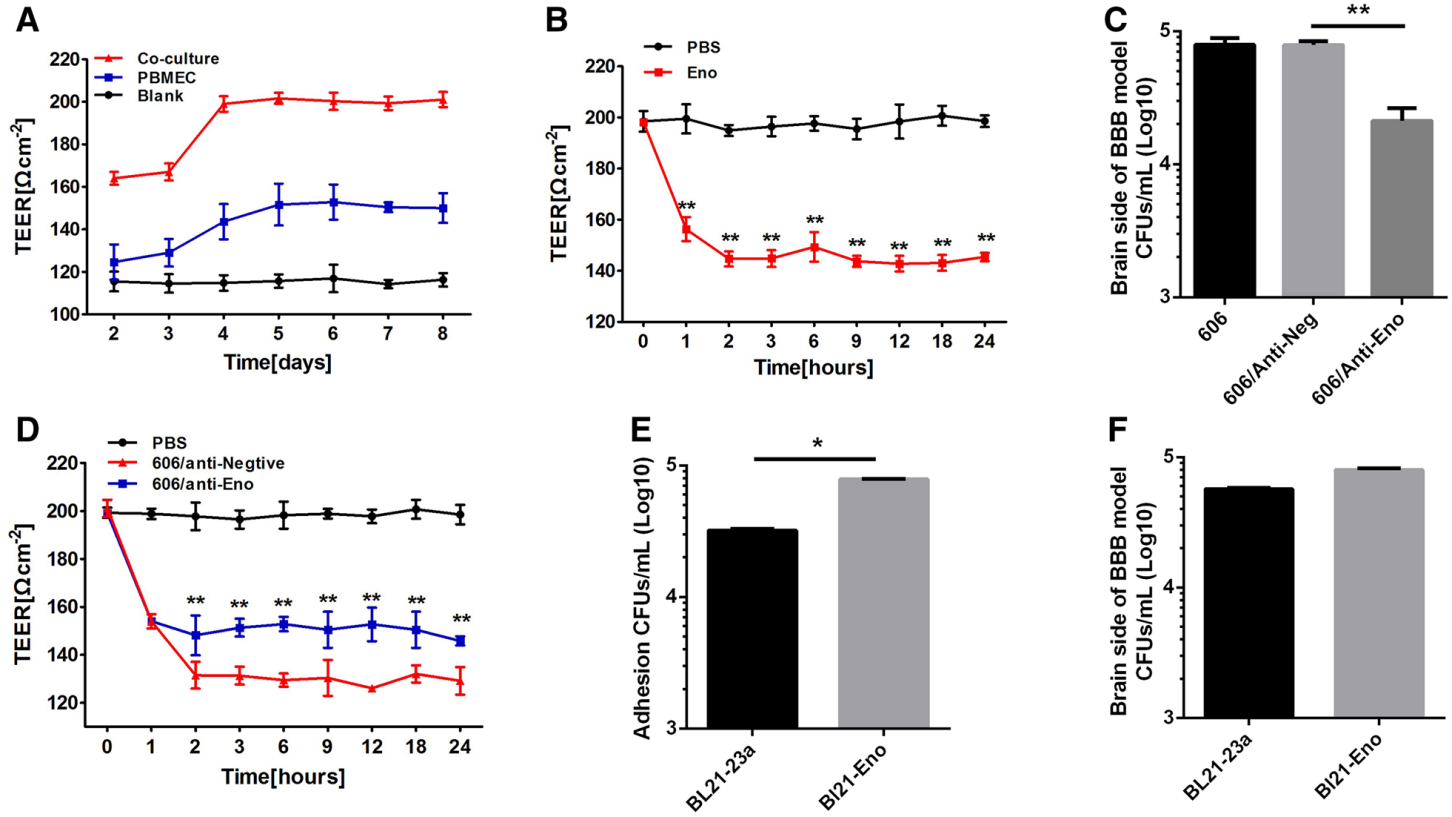
**Eno destroys BBB model integrity**. **A** co-culture BBB model was established successfully; **B** Eno disrupts BBB integrity based on TEER; **C** Rabbit anti-Eno IgG inhibits SS2 penetration into the BBB; **D** Effect of anti-Eno on the TEER of the SS2-treated BBB model; **E**, **F**, BL21-Eno has stronger adhesion to and invasion into PBMECs and has a stronger ability to cross the BBB than does BL21-23a (*n* = 3); (606 = SS2 strain CVCC606, anti-Eno = rabbit anti-Eno IgG, anti-Negative = isotype IgG control, i.e., same as anti-Eno, ^*^p < 0.05, ^**^p < 0.01, ^NS^*p* > 0.05).

**Figure 2:**
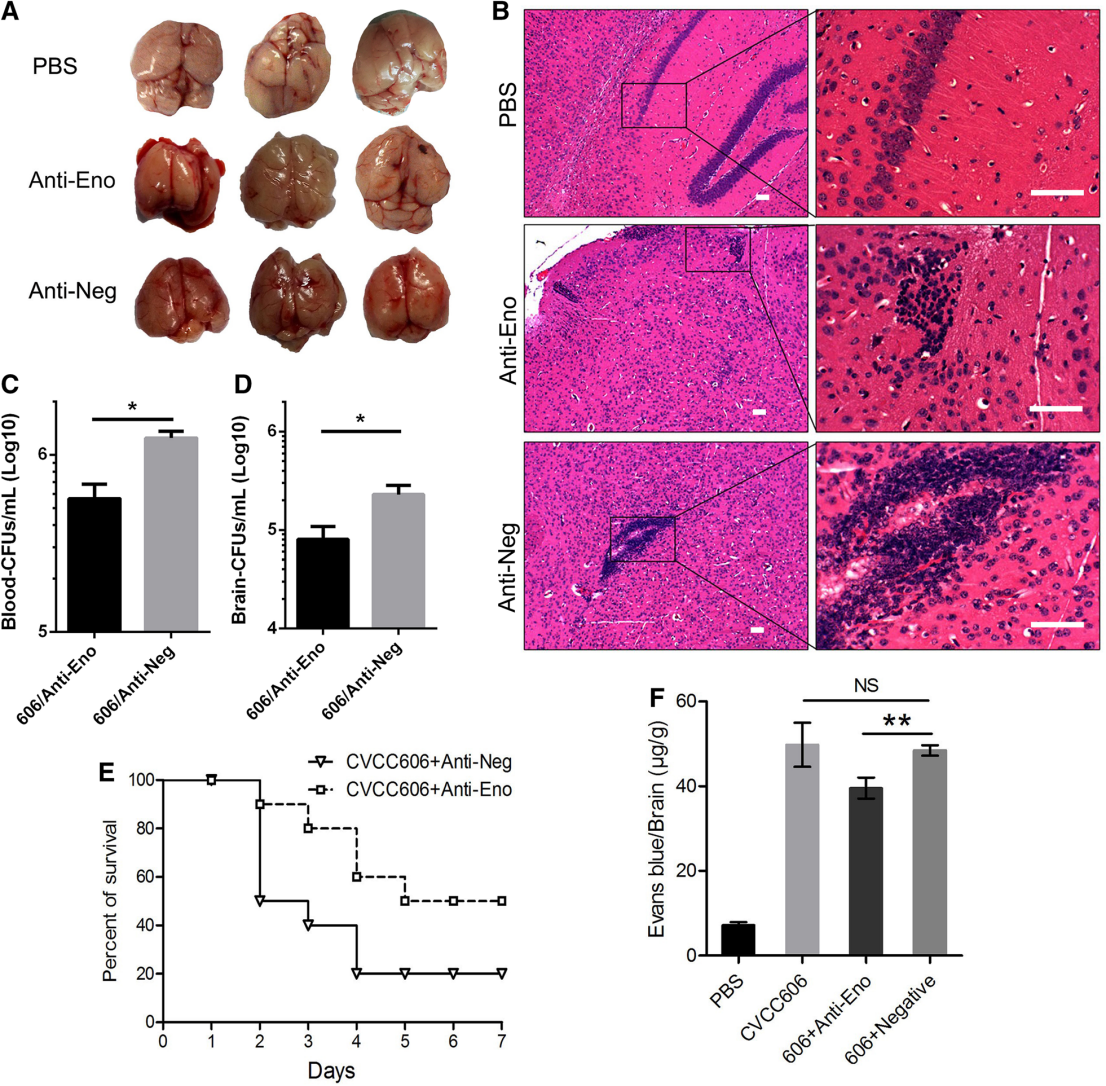
**Eno and mediates SS2 invasion of the brain**. **A** Haemorrhage in the brains of mice pre-injected with anti-Eno was weaker compared with that of the control group; **B** Brains of mice pre-injected with anti-Eno showed weaker immune-cell infiltration compared with that of the control group (Scale bar 100 μm); **C, D** Blocking Eno with anti-Eno resulted in decreased bacterial loads in both the blood and brains of CVCC606-infected mice; **E** Blocking Eno increased the survival rate of SS2-infected mice; **F** Blocking Eno reduced SS2-caused EB into the brain (*n* = 10).

The co-culture BBB model was established successfully based on TEER measurement (Figure 1A) according to a previous study [16]. The addition of Eno to the blood side of the BBB model resulted in, compared with the PBS control group, a rapid decrease (within 1 h) in TEER (Figure  1B). Anti-Eno decreased SS2 invasion into the BBB model (Figure  1C) and weakened CVCC606-induced BBB damage based on TEER monitoring (Figure  1D). Additionally, the ability of BL21-Eno (BL21-pET23a::Eno) to adhere to PBMECs (Figure  1E) and cross the BBB (Figure  1F) was stronger than that of BL21-23a (BL21-pET23a). These results confirmed that Eno plays an important role in SS2 adhesion to PBMECs and penetration into the BBB. Compared with the control group, there was less haemorrhage in SS2-infected mouse brains pre-injection with anti-Eno (Figure  2A). HE staining showed less immune-cell infiltration in mouse brains pre-injected with anti-Eno compared with that of the control group (Figure  2B). The nucleus of infiltrating cells are deeply stained and no nucleolus. The shape of cells are irregular round and cytoplasm are rarely seen. It was conducted the infiltrating cells are mainly monocyte macrophage (Figure  2B). Blocking Eno resulted in a decreased bacterial load in the blood and brain (Figure  2C, D) and increased the survival rate of SS2-infected mice (Figure  2E). This also resulted in a reduction in Evans Blue (EB) permeability to the brain (Figure  2F). Collectively, these results suggest that Eno plays an important role in disruption of the BBB by SS2.

### Eno induces apoptosis in PBMECs and promotes SS2 BBB penetration

**Figure 3:**
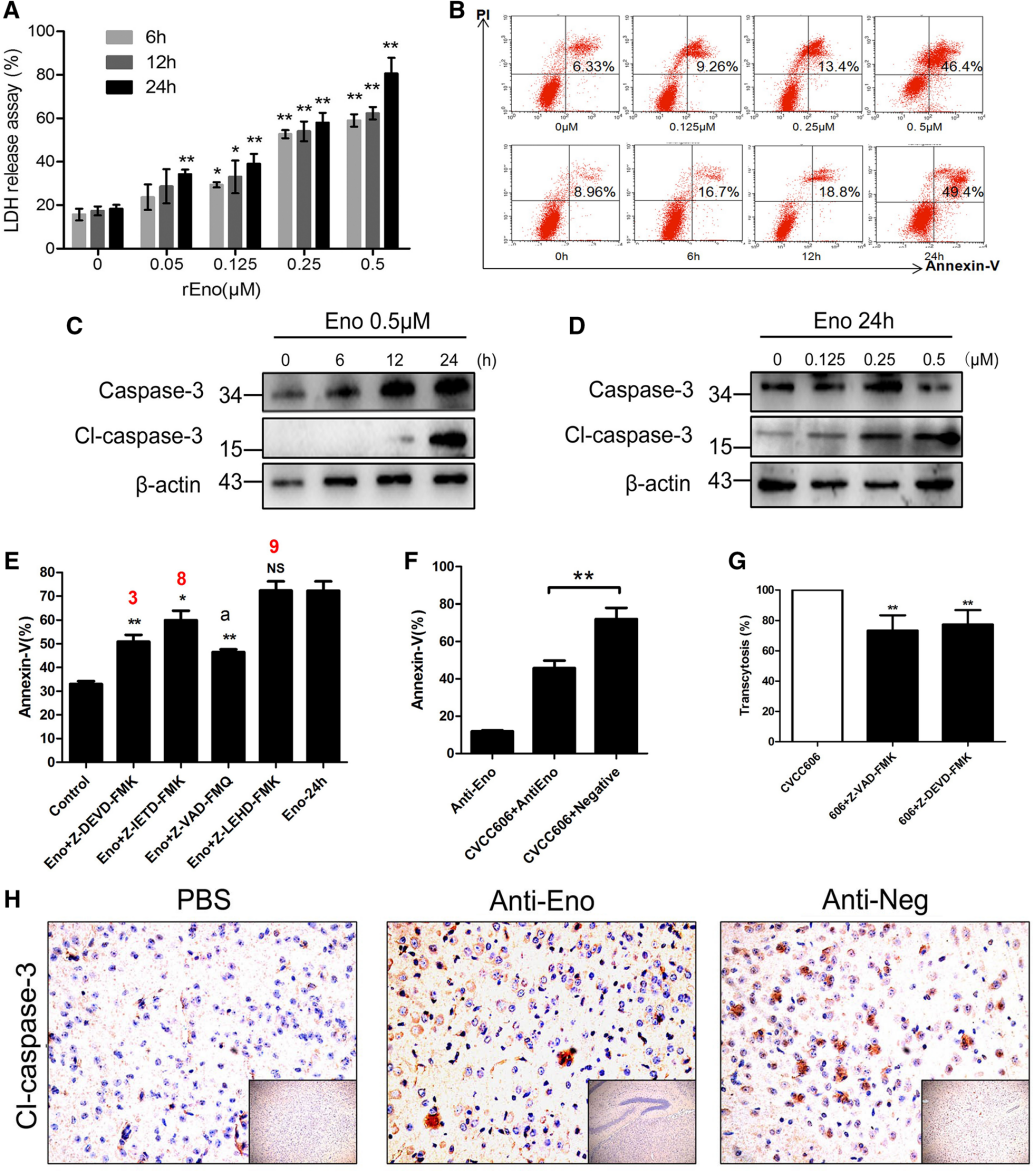
**Analysis of Eno-induced cell apoptosis**. **A** Eno-induced cytotoxicity for PBMECs showed a time-dependent enhancement; **B** Eno-induced apoptosis for PBMECs showed time- and dose-dependent enhancement by flow cytometry; **C, D** Eno-induced cleaved caspase-3 expression showed time- and dose-dependent enhancement by Western blotting; **E** Eno-induced apoptosis is dependent on caspase-8 and caspase-3 activation; Z-DEVD-FMK is a caspase-3 inhibitor; Z-ITED-FMK is a caspase-8 inhibitor; Z-LEHD-FMK is a caspase-9 inhibitor; a, Z-VAD-FMQ is an inhibitor for all caspases; **F** Blocking Eno of SS2 by Rabbit anti-Eno IgG can inhibit SS2-induced apoptosis. The role of Eno in SS2-induced PBMEC apoptosis; **G** The role of apoptosis in SS2 breaking into the BBB model; **H** Immunohistochemical analysis of mouse brains showed that injection with rabbit anti-Eno inhibited cleaved caspase-3 expression caused by SS2 infection (scale bar, 100 μm). (^*^p < 0.05, ^**^p < 0.01, ^NS^*p* > 0.05).

Eno induced cytotoxicity in a time- and dose-dependent manner as adjudged by LDH release assays (Figure  3A) and promoted PBMEC apoptosis based on Annexin-V/PI staining (Figure  3B) using the Annexin V-FITC Apoptosis Detection Kit (Sigma, China, APOAF) and immunoblotting for caspase-3 (Figure  3C, D). Eno also induced apoptosis in both hCMEC/D3 (Additional file 1: Figure  S1A) and 293T cells (Additional file 1). The addition of a caspase inhibitor (Z-VAD-FKQ) and caspase-3 (Z-DEVD-FMK) and 8 (Z-IETD-FMK) inhibitors reduced Eno-induced apoptosis (Figure  3E). Treatment of SS2 with anti-Eno inhibited SS2-induced apoptosis compared with the control group (Figure  3F). Caspase inhibitors (Z-VAD-FMK and Z-DEVD-FMK), used to block Eno-induced apoptosis, inhibited SS2 invasion in the BBB model (Figure  3G). ICR mice were injected with anti-Eno prior to SS2 infection. The immunohistochemistry results showed that, compared with the isotypic IgG-injected group, cleaved caspase-3 in the brains of SS2-infected mice exhibited lower expression (Figure  3H). These results indicate that SS2 invasion of the BBB requires, at least partially, Eno-induced apoptosis.

### Eno interacts with RPSA on the cell surface, inducing host-cell apoptosis

**Figure 4:**
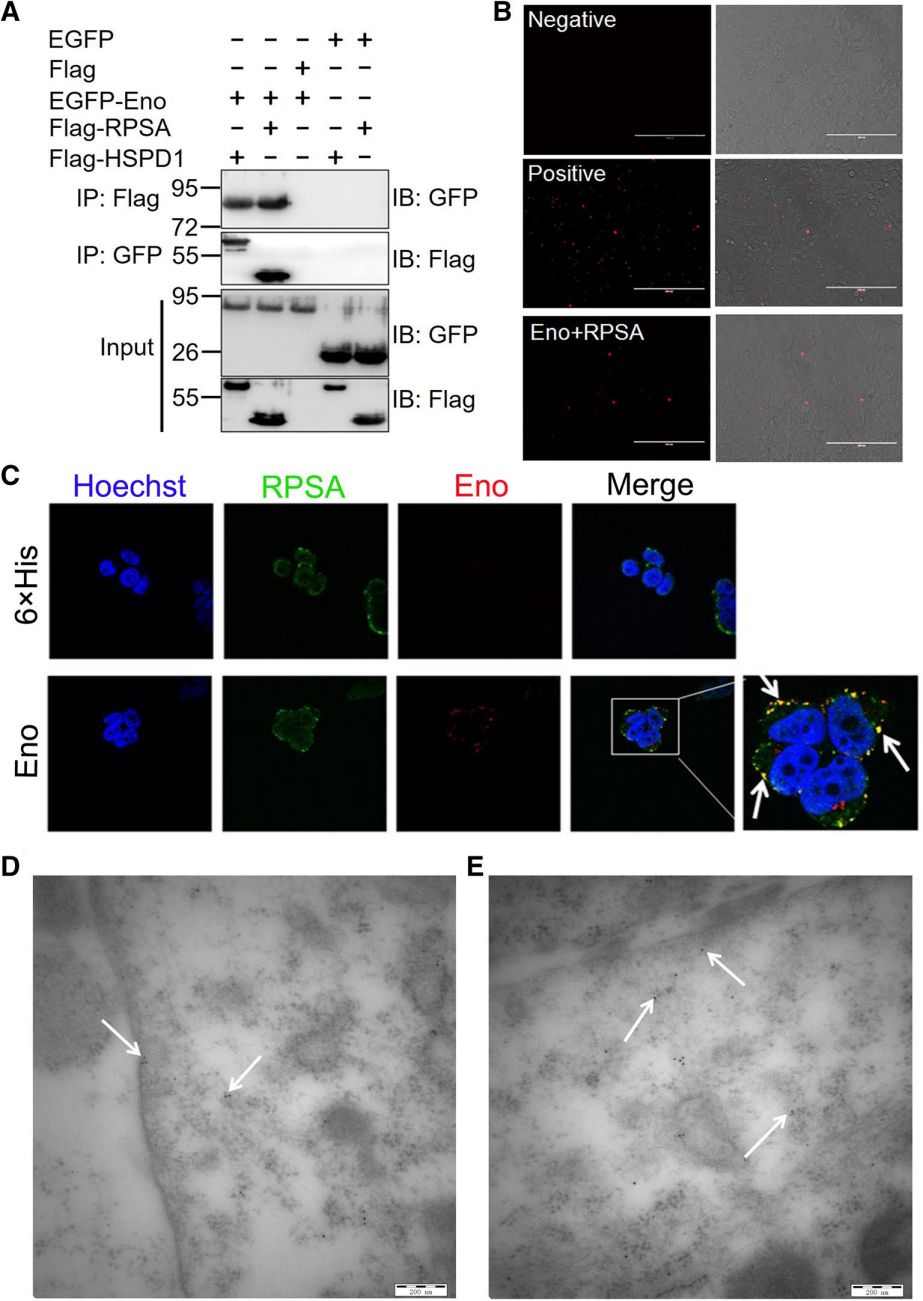
**Eno interacts with RPSA on the cell surface**. **A** Eno-RPSA interaction shown by Co-IP; **B** Eno-RPSA interaction shown by BiFC (scale bar, 200 μm); **C** Eno and RPSA were co-located on the cell surface by immunofluorescence analysis (scale bar, 10 μm); **D, E** Immunoelectron microscopy analysis of Eno-RPSA interaction in Eno-treated hCMEC/D3. The arrow indicates co-located Eno (labelled with 5-nm gold particles) and RPSA (labelled with 10-nm gold particles) on the cell surface (**D**) and in the cytoplasm (**E**) (scale bar, 200 nm).

**Figure 5:**
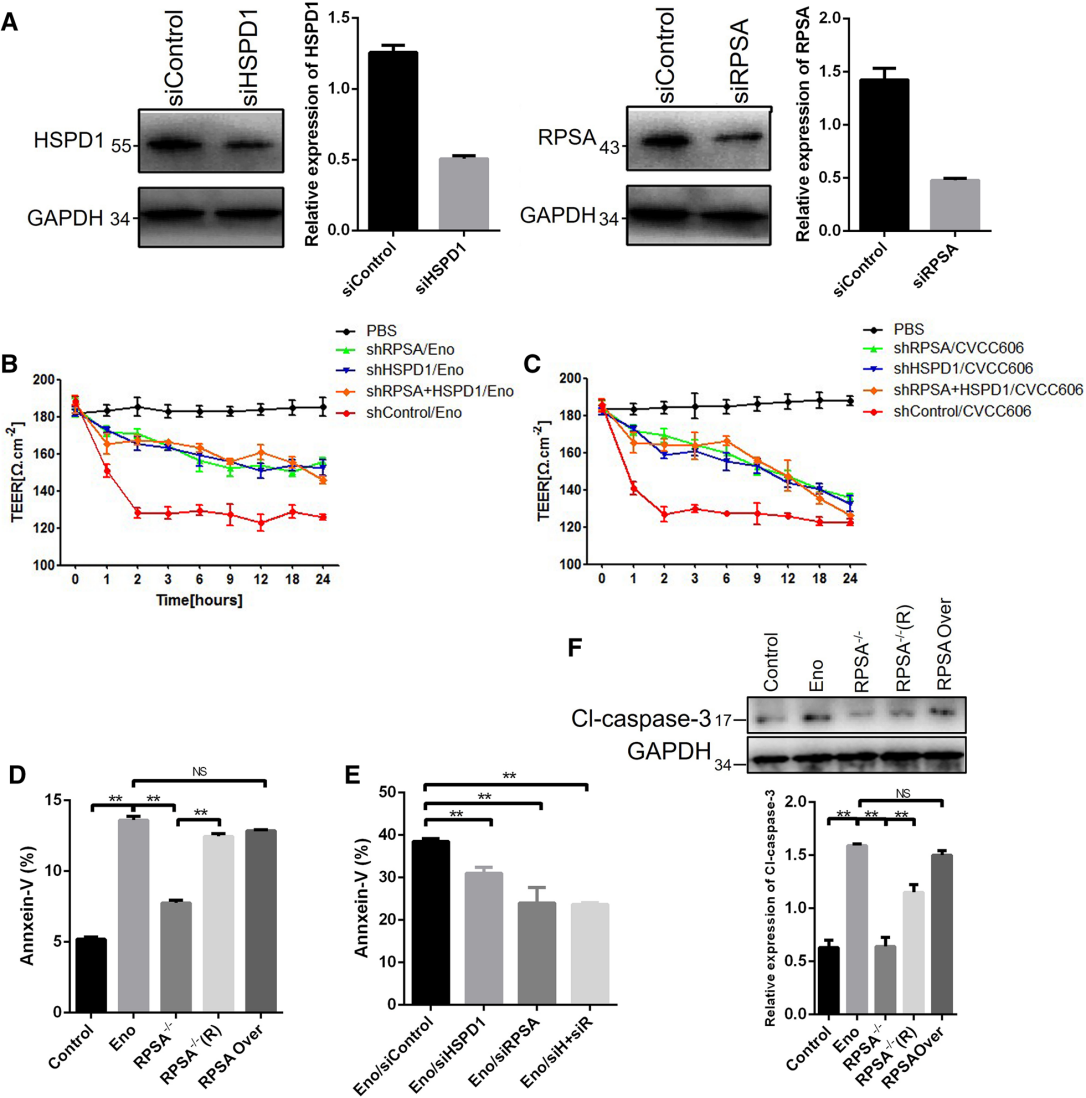
**RPSA mediates Eno-induced apoptosis and BBB damage**. **A** Knockdown of HSPD1 and RPSA in PBMECs after transfection with siRNA RPSA and/or HSPD1; **B** Knockdown of RPSA inhibited BBB damage induced by Eno based on TEER; **C** Knockdown of RPSA inhibited BBB damage induced by SS2 based on TEER; **D** Knockdown of RPSA inhibited PBMEC apoptosis induced by Eno based on flow cytometry of Annexin V/PI-stained PBMECs; **E** Knockout of RPSA inhibited 293T cell apoptosis induced by Eno; **F** Knockout of RPSA inhibited 293T cell apoptosis induced by Eno based on Western blotting analysis for activated caspase-3. (^*^p < 0.05, ^**^p < 0.01, ^NS^*p* > 0.05).

Pull-down assays were carried out using Eno as the bait protein. There were clear differences in protein bands between His-Eno and His tag groups (Additional file 1: Figure  S2A), which were analysed by LC–MS/MS. Thirteen proteins with a predicted cell surface location were selected (Additional file 1: Figure S2B, Additional file 1: Table S1). Then, 13 genes were cloned into the pDisplay vector, transfected into 293T cells, and analysed by flow cytometry. RPSA and HSPD1 proteins were identified to interact with Eno (Additional file 1: Figure  S2C). Eno clearly interacted with RPSA in Co-IP (Figure  4A). pbEno-Myc-LC151 and pbRPSA-HA-KN151 plasmids were co-transfected into 293T cells and analysed by the bimolecular fluorescence complementary (BiFC) assay. The combined Eno and RPSA were in close proximity as adjudged by active red fluorescence (Figure  4B), confirming that Eno interacts with RPSA (Figure  4B) but not HSPD1 (data not shown). Immunofluorescence analysis indicated that Eno and RPSA co-located on the cell surface, as indicated by yellow (merged) spots (Figure  4C, white arrows). Immunoelectron microscopy results also showed that Eno (labelled with 5-nm gold particles) and RPSA (labelled with 10-nm gold particles) existed nearly in pairs in the cytoplasm and on the membrane of cells (Figure  4D and E). Knockdown of RPSA (Figure  5A) inhibited Eno- or SS2-induced BBB damage (Figures 5B and 4C). Overexpression of RPSA had no effect on Eno-induced apoptosis in 293T cells (Figure  5F), but knockout of RPSA significantly reduced Eno-induced apoptosis (Figure  5D-F). Knockout of RPSA significantly inhibited Eno-induced apoptosis, and this was reversed by RPSA complementation (Figure  5D-F).

Although HSPD1 cannot bind with Eno, knockdown of HSPD1 (Figure  5A) also inhibited Eno- or SS2-induced BBB damage (Figure  5B, C) and apoptosis (Figure  5D). Taken together, these results suggest that RPSA can bind Eno to initiate host-cell apoptosis and that HSPD1 also plays an important role in Eno-induced apoptosis without binding to Eno.

### HSPD1 expression promotes host-cell apoptosis regulated by Eno binding to RPSA through p38/ERK MAPK-eIF4E signalling

**Figure 6:**
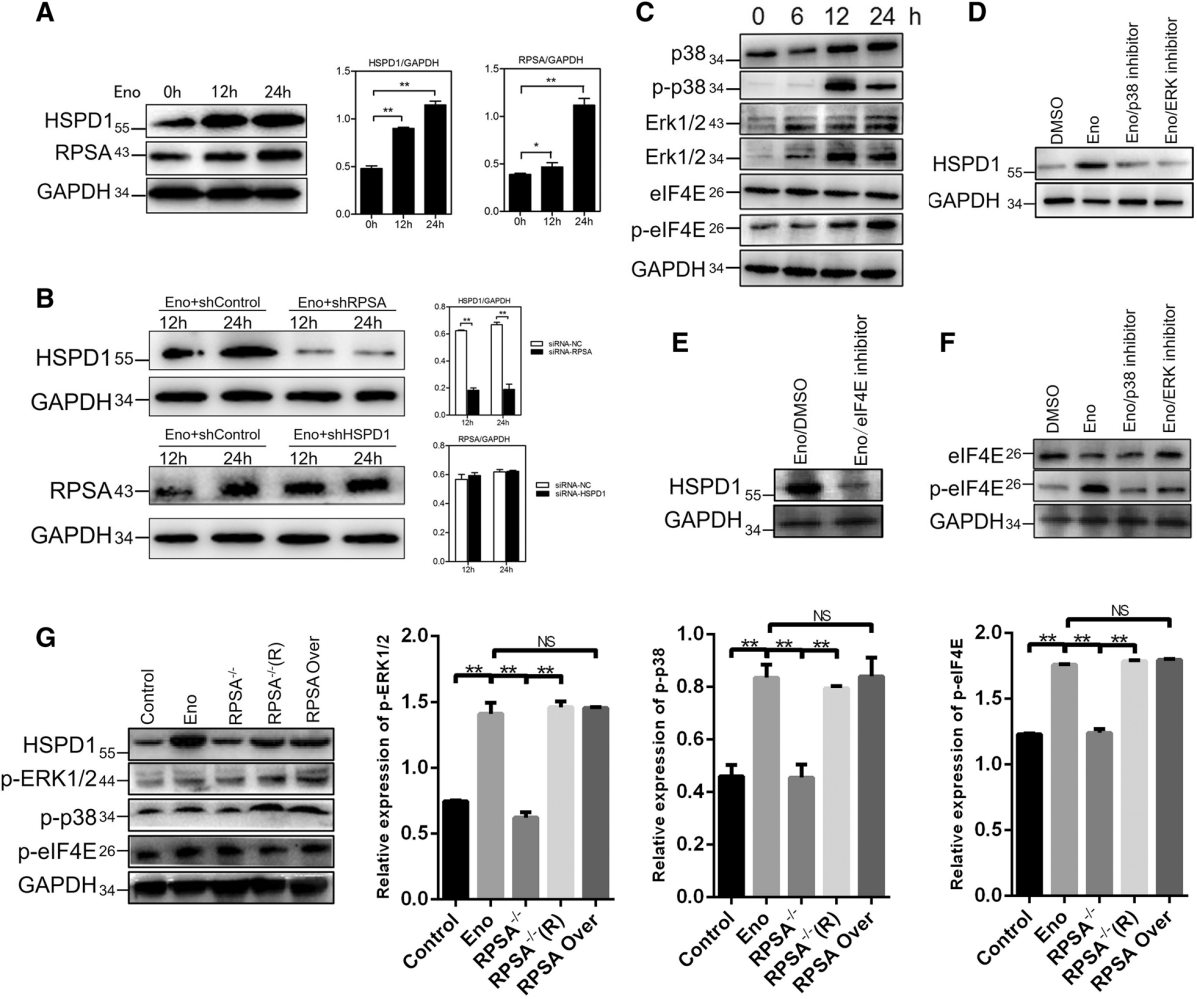
**Eno activates p38/ERK MAPK-eIF4E signalling to increase HSPD1 expression by binding to RPSA**. **A** Eno treatment increased expression of RPSA and HSPD1 in PBMECs; **B** Knockdown of RPSA decreased Eno-promoted HSPD1 expression, but knockdown of HSPD1 did not decrease Eno-promoted RPSA expression; **C** Western blotting analysis of p38, ERK, and eIF4E of Eno-treated cells; **D** Analysis of MAPK activation and its effect on HSPD1 expression in Eno-treated cells; **E** Analysis of HSPD1 expression in Eno-treated cells after inhibition of eIF4E; **F** Analysis of eIF4E activation in Eno-treated cells after inhibition of MAPK signalling; **G** The effect of RPSA knockout or overexpression on Eno-induced MAPK-signalling activation in cells. RPSA-/-(R): Complementary RPSA; RPSA (Over): RPSA overexpression. (*p < 0.05, **p < 0.01, ^NS^*p* > 0.05).

Increased expression of both RPSA and HSPD1 was detected in an Eno-treated, time-dependent manner (Figure  6A). Knockdown of RPSA inhibited the increased HSPD1 expression in Eno-treated PBMECs, whereas knockdown of HSPD1 had no effect on RPSA expression (Figure  6B). These results suggested that Eno-RPSA interaction promoted HSPD1 expression, which in turn promoted PBMEC apoptosis. Eno-RPSA interaction promoted the expression of HSPD1 which the both intracellular and extracellular HSPD1 was increased in process of Eno induced apoptosis (Additional file 1: Figure  S3A). But extracellular HSPD1 induces apoptosis (Additional file 1: Figure  3C and D) through Toll like receptor 4 (TLR4) has been clarified in a previous study [27, 28]. In order to identify the signalling pathway(s) activated by Eno-RPSA interaction, phosphor-proteomics (carried out by QL-bio Co., Ltd) were used to identify cytoplasmic phosphorylated proteins in Eno-treated PBMECs. The phosphorylation levels of ten members of the MAPK family present in the cytoplasm of Eno-treated PBMECs were differentially expressed compared with those of the non-stimulated group (Additional file 1: Figure  S4A). Immunoblotting showed that the phosphorylation levels of p38 and ERK increased after Eno treatment in PBMECs, while that of JNK did not change (Figure  6C). The addition of SB203580 and PD098059, which inhibit p38 and ERK, respectively, prevented the Eno-promoted expression of HSPD1 (Figure  6D). A nuclear proteomics analysis (carried out by QL-bio Co., Ltd) of PBMECs showed that three molecules of the eIF family (EIF4E2, EIF3J, and EIF4A2) were highly expressed in nucleoproteins in Eno-treated PBMECs (Additional file 1: Figure  4B). Phosphorylation of eIF4E was increased in Eno-treated PBMECs (Figure  6F). Eno-induced HSPD1 expression was inhibited by eIF4E inhibitor (SBI-0640756) (Figures  6E and G). To determine whether Eno-induced activation of p38 and ERK is regulated by RPSA, RPSA was overexpressed or knocked down by siRNA in Eno-treated 293T cells. Knockdown of RPSA resulted in inhibition of Eno-induced p38 and ERK activation, although overexpression of RPSA had no effect on the activation of p38 or ERK (Figure  6H). Knockdown of RPSA inhibited the Eno-induced activation of eIF4E (Figure  6H). Inhibiting phosphorylation of p38 and ERK with corresponding inhibitors also inhibited the Eno-induced activation of eIF4E (Figure  6G). These results suggest that Eno-RPSA interaction promoted the activation of p38/ERK MAPK-eIF4E signals, which in turn promoted HSPD1 expression.

### PBMEC apoptosis induced by Eno binding to RPSA disrupts the BBB in vivo

**Figure 7:**
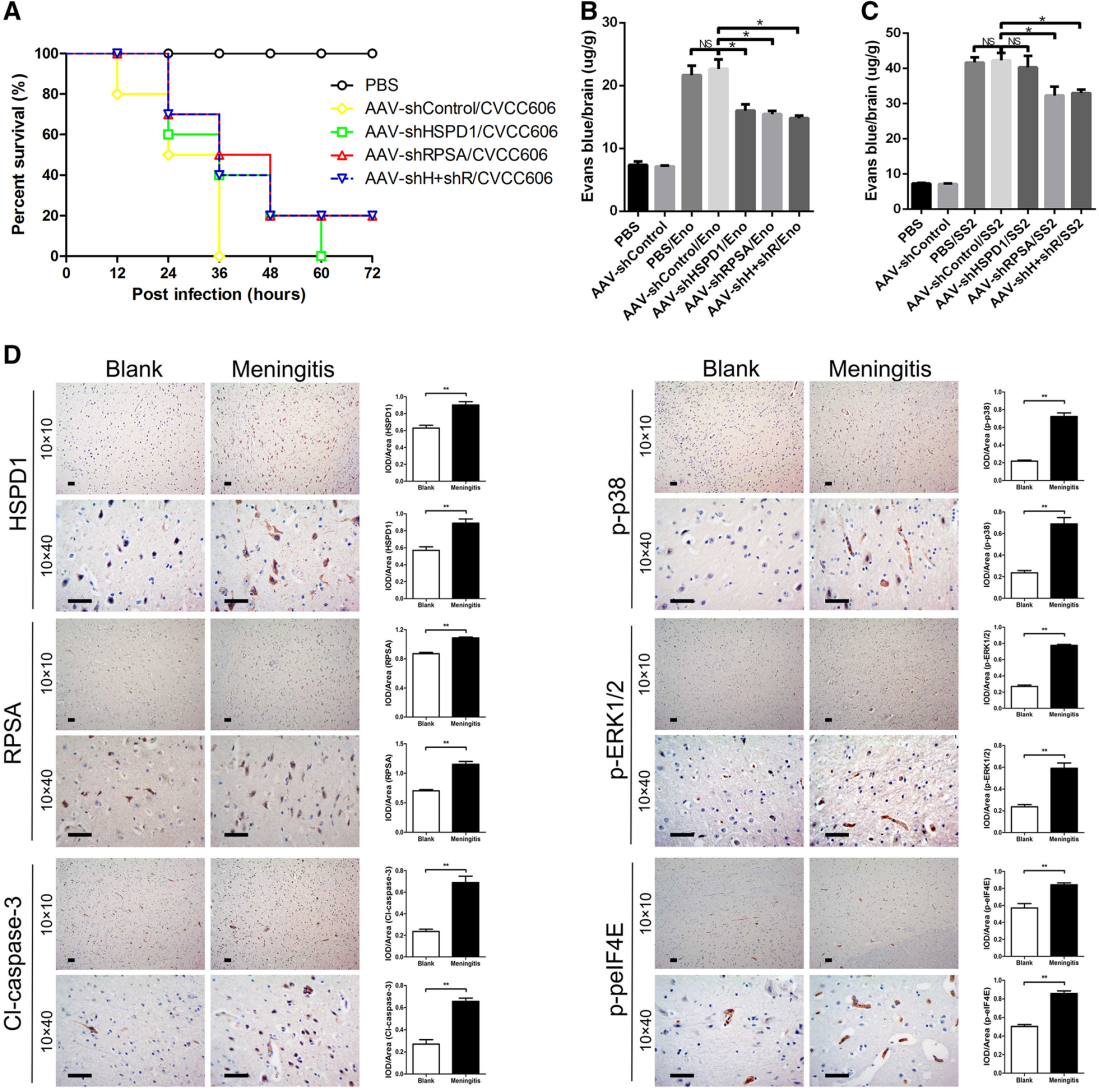
**Eno disrupts the BBB in vivo by inducing PBMEC apoptosis**. **A**, Survival rates of SS2-infected mice; **B** Eno enhanced EB permeability into mouse brains; **C** SS2 enhanced EB permeability into mouse brains; **D** SS2 meningitic pig brains have higher expression of apoptosis-related molecules compared with that of healthy pig brains by immunohistochemical analysis; (*p < 0.05, **p < 0.01) (scale bar, 100 μm).

Next, RPSA and HSPD1 expression were knocked down in ICR mouse brains using adeno-associated virus (AAV2/9) carrying RPSA and HSPD1 shRNA (Additional file 1: Figure  5A and B). ICR mice were treated with Eno (2 µg) or infected with SS2 (10^5^ CFUs) via the tail vein. The survival rates of SS2-infected mice with knockdown of RPSA (50%) and HSPD1 (40%) were significantly increased at 36 h post-infection (shControl group, 0%) (Figure  7A). EB is a dye that cannot enter the brain via the BBB under normal circumstances, and can be used to evaluate the integrity of the BBB [29]. EB permeability experiments showed that knockdown of PRSA and HSPD1 reduced EB permeability (Figure  7C) in SS2-infected mouse brains, and knockdown of RPSA weakened Eno-induced BBB damage (Figure  7B). These results suggest that RPSA and HSPD1 mediate Eno- and SS2-caused brain damage. Immunohistochemistry also confirmed that knockdown of RPSA and/or HSDP1 inhibited Eno-induced activation of caspase-3, p38, ERK, and eIF4E 24 h post-Eno-injection. The expression of eIF4E was also consistent with the in vivo results (Additional file 1: Figure  S6).

The Bama miniature piglets were infected with SS2 JZLQ022, a clinical isolate from a porcine meningitis case. Hyperpyrexia was monitored, and the body temperature of some pigs infected with JZLQ022 increased to 40 °C at 24 h. At 4 days post infection, some pigs had difficulty walking, and at 5 days foaming at the mouth and ataxia were observed. The results were in line with the SS2 meningitis model that we had previously successfully established [25]. By autopsy, conspicuous macroscopic lesions, including lung purulent lesions, swelling and infarction of the spleen, and kidney nephremia, were observed in the JZLQ022 infection groups. Eno induced apoptosis was detected by Immunohistochemistry which showed that RPSA, HSPD1, activated caspase-3, phosphorylated p38 and ERK, and phosphorylated eIF4E were higher in meningitic pig brains than in the control group (Figure  6D). These results further verified the molecular mechanism by which Eno induces apoptosis.

## Discussion

Eno is a relatively conservative protein and has long been considered an ancient single-function catalytic enzyme by researchers. In SS, the native protein (designated SS Eno) possesses not only high homology with other bacterial enolases but also enolase activity. However, in recent years, some bacterial Enos have been found to be important for virulence [16, 30]. For example, *Streptococcus pneumoniae* (*S. pneumonia*) Eno induces the formation of Neutropil Extracellular Traps (NETs) [31] facilitating evasion of complement killing by interacting with C4b-binding proteins [32]. Eno from Streptococcus canis (S. canis) interacts with host plasminogen to promote bacterial viability in vivo. The present group’s other work [16, 17] has suggested a role for SS Eno in BBB impairment, and in this study, the mechanism was sought. This study reveals for the first time that binding of SS2 to host-cell RPSA induces higher HSPD1 expression, which initiates apoptosis resulting in damage to BBB integrity, and entry of the bacterium into the CNS. Thus our study increases our understanding of the pathogenesis of SS2, and may provide a novel strategy of meningitis control, for example, through blocking the interaction of Eno with RPSA and inhibition of apoptosis.

Fibronectin (Fn) and plasmin (pFg) have been described as receptor proteins of Eno, and interactions can facilitate escape from phagocytosis and complement-mediated killing [15, 33]. However, this study found that the addition of non-specific serum did not affect Eno-induced PBMEC apoptosis, indicating that apoptosis induced by Eno was independent of Fn and pFg (data not shown). Therefore, it was suspected that Eno may bind to an unknown molecule on the cell surface and mediate apoptosis. Therefore, we suspected that Eno may bind to an unknown molecule on cell surface and then mediate apoptosis. Interestingly, and RPSA identified as one of Eno-binding proteins beside Fn and pFg. And the interaction of SS2 Eno with RPSA induced apoptosis of PBMECs.

RPSA is also known as laminin receptor (LAMR) since it binds laminin. RPSA is found in many kinds of cells with a wide range of cellular locations, including cell membranes, ribosomes, and nuclear membranes, and is expressed in two forms: 37LR and 67LR [18]. *E. coli* K1 is an important cause of meningitis, with cytotoxic necrosis factor 1 (CNF1) being considered a major virulence determinant. It has been found that the 37-kDa laminin receptor precursor (37LRP) is the receptor of CNF1 [34, 35]. However, the molecular mechanism of adhesion and invasion of bacteria and the destruction of the BBB mediated by RPSA is still unclear. This study revealed that Eno enhances BBB permeability by binding to RPSA and confirmed that this interaction plays an important role in BBB penetration both in vitro and in vivo. Passive protection of mice with anti-Eno antibody increased survival, indicating that Eno-RPSA interaction is important for SS2 meningitis.

**Figure 8:**
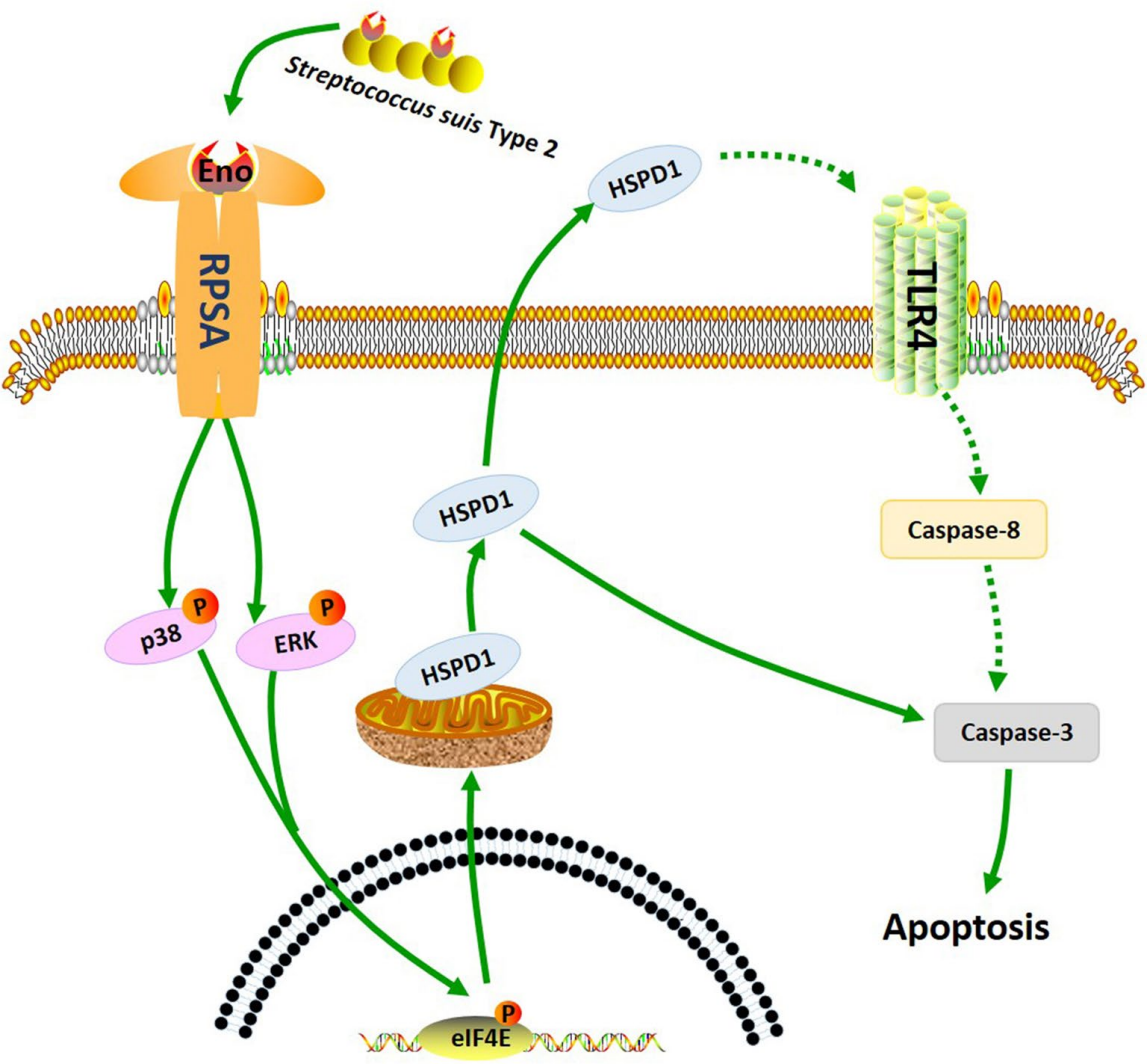
**The signalling pathway of Eno-RPSA induced the loss of BBB integrity**. SS2 Eno interacts with host RPSA, resulting in activated p38/ERK and MAPK-eIF4E signalling and, in turn, promoting expression of HSPD1 followed by caspase -3-induced apoptosis. (Green arrows = activation. Red arrows = inhibition).

In conclusion, SS2 Eno interacts with RPSA on the PBMEC surface, inducing the p38 and ERK pathways to activate eIF4E and resulting in HSPD1 expression followed by caspase-3 mediated apoptosis (Figure  8). The net result is increased permeability of the BBB and increased adherence and invasion of SS2 into the CNS, facilitating meningitis. Host pathways targeted by the virulence factor SS2 Eno have been elucidated, allowing for the identification of new therapeutic strategies to protect the host against SS2 meningitis.

## Supplementary Information

Flow cytometry analysis of Eno-induced apoptosis. **A** Eno induced hCMEC/D3 apoptosis by flow cytometry in 24 h; **B** Eno induced 293T- cell apoptosis by flow cytometry in 24 h.

Screening and identification of Eno binding proteins in PBMECs based on LC/LC-MS and flow cytometry analysis. **A**, **B** Thirteen different proteins in protein bands between His-Eno and His tag groups were found by IP and LC/LC-MS; **C** pDisplay was a eukaryotic cell membrane-displayed vector. 293T cells were transfected with pDisplay::HSPD1, RPSA, JUP, TPM4, G3BP1, PTBP1, PGAM1, HH4, HSP90AB, RPS5, PHB, RPL11, and MYO1C, respectively. After 24 h, recombinant His-Eno was added to the culture dish to adhere the transfected cells. Mouse anti-His IgG was used, and flow cytometry was carried out to analyse the adhesion rate of Eno. Eno showed stronger adhesion to cells displaying RPSA and HSPD1 compared with that of the other experimental group and vector group (labeled “untreated”). We found Eno promoted HSPD1 secretion in PBMECs at 12 h by Enzyme Linked Immunosorbent Assay (ELISA). Knockdown RPSA inhibited HSPD1 secretion induced by Eno. In order to detect the effect of extracellular increased HSPD1 on PBMECs apoptosis. Experiments were designed as followed. The experiment was divided into five groups (*n* = 3). Remove Sup: PBMECs were treated by 0.5 uM Eno for 12 h and culture supernatant were removed. Fresh complete medium was added and cultured for another 12 h. antiE+antiH: PBMECs were treated by 0.5uM Eno for 12 h. Eno and HSPD1 antibodies was added and PBMECs were cultured for another 12 h. antiE: PBMECs were treated by 0.5 uM Eno for 12 h and Eno antibody was added and cultured for another 12 h. Enolase: PBMECs were treated by 0.5 uM Eno for 24h and analysed by flow cytometry. AntiControl: Negative isotype rabbit IgG was added into per well. All groups PBMECs were collected and analysed by flow cytometry.

Increased HSPD1 secretion induced by Eno promote PBMEC apoptosis. **A** Eno promote secretion ofHSPD1. **B** RPSA mediate Eno induced Increased HSPD1 secretion by RNAi assay. **C** and **D** Flow cytometry was used for extracellular increased HSPD1 promote PBMEC apoptosis. AntiControl, isotype rabbit IgG with Eno antibody/HSPD1 antibody; antiE, Eno antibody; antiH, HSPD1 antibody; Remove sup, culture supernatant were removed at 12 h post treated by Eno.

Screening of the signalling pathway(s) activated by Eno-RPSA interaction based on proteomics analysis. **A** Cytoplasmic phosphorylated proteomics analysis of Eno-stimulated PBMEC for 24 h; **B** Nuclear proteomics analysis of Eno-stimulated PBMEC for 24 h. EIF4A2, EIF4E2, and EIF3J, the EIF family proteins, were increased in Eno-treated PBMECs (indicated as red) compared with the untreated PBMEC control (indicated as grey).

Construction of the mouse model with knockdown RPSA and/or HSPD1 in the brain. **A** Two months after the third ventricle injection, frozen slices of the brain showed that AAV, which carried mCherry-RPSA or GFP-HSPD1, is distributed throughout the brain. Control AAV-shRNA (shControl) carried GFP (scale bar, 2000 μm); **B** Western blotting showed that the knockdown RPSA and/or HSPD1 model of the mouse brain was successfully established two months after the third ventricle injection by AAV, which carried mCherry-RPSA or GFP-HSPD1.

Verification of mechanism of Eno induced apoptosis in mice. Intravenous injection of Eno or SS2 promoted the expression of apoptosis-related molecules in mouse brains by immunohistochemical analysis (*p < 0.05, **p < 0.01) (scale bar, 100 μm).

13 Proteins suspected to interact with Eno.

siRNA used for RPSA and HSPD1 knockdown.

## Data Availability

Not applicable.

## Abbreviations

Eno: Enolase
RPSA: 40S Ribosome protein SA
BBB: Blood brain barrier
HSPD1: Heat-shock protein family D member 1
PBMECs: Porcine brain microvascular endothelial cells
hCMEC/D3: Human brain microvascular endothelial cell line
ACs: Astrocytes
RNAi: RNA interference
HE: Hematoxylin-eosin
GFAP: Glial fibrillary acidic protein
TEER: Transendothelial electrical resistance
EB: Evans blue
