# Supplementary figures and images for: Streptococcus suis serotype 2 enolase interaction with host brain microvascular endothelial cells and RPSA-induced apoptosis lead to loss of BBB integrity
Source: Vet Res. 2021 Feb 22;52:30. doi: 10.1186/s13567-020-00887-6 (PMC7898445; doi:10.1186/s13567-020-00887-6)

**
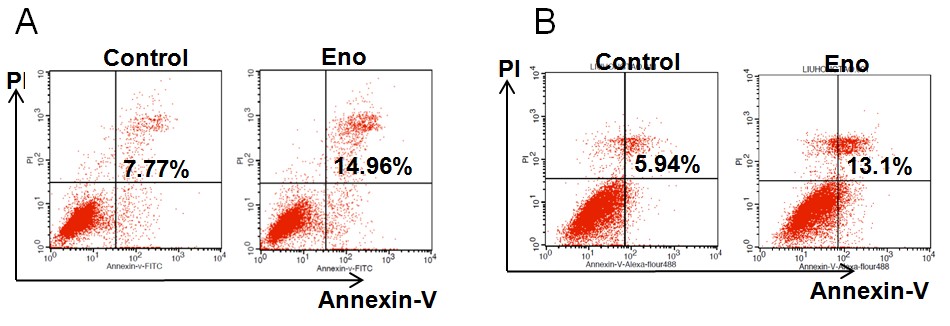
**

Supplement: Supplementary file 1 — Flow cytometry analysis of Eno-induced apoptosis. A Eno induced hCMEC/D3 apoptosis by flow cytometry in 24 h; B Eno induced 293T- cell apoptosis by flow cytometry in 24 h. [file 13567_2020_887_MOESM1_ESM.docx]

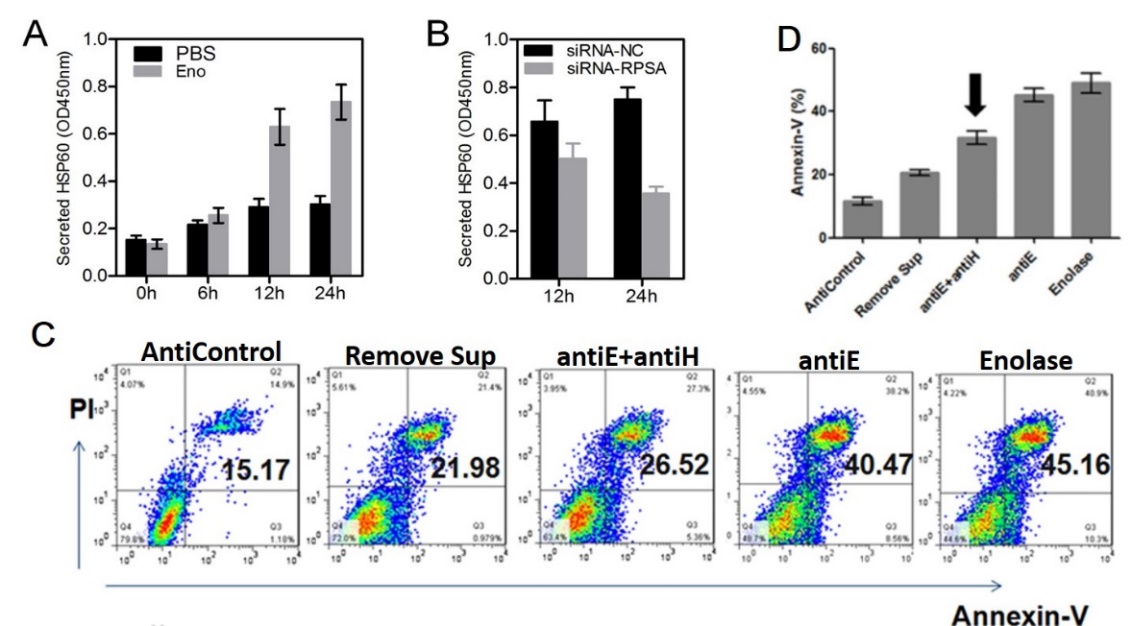

Supplement: Supplementary file 3 — Increased HSPD1 secretion induced by Eno promote PBMEC apoptosis. A Eno promote secretion ofHSPD1. B RPSA mediate Eno induced Increased HSPD1 secretion by RNAi assay. C and D Flow cytometry was used for extracellular increased HSPD1 promote PBMEC apoptosis. AntiControl, isotype rabbit IgG with Eno antibody/HSPD1 antibody; antiE, Eno antibody; antiH, HSPD1 antibody; Remove sup, culture supernatant were removed at 12 h post treated by Eno. [file 13567_2020_887_MOESM3_ESM.docx]

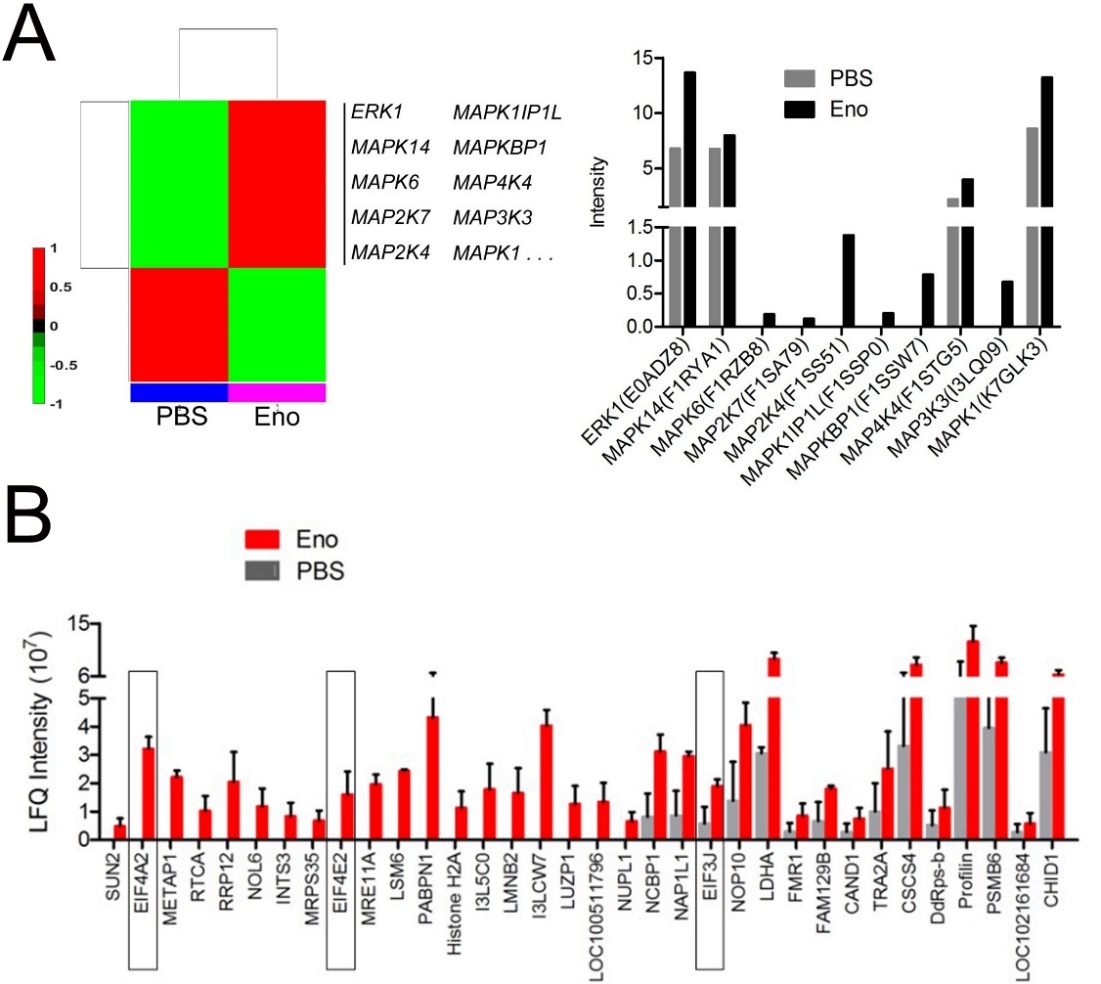

Supplement: Supplementary file 4 — Screening of the signalling pathway(s) activated by Eno-RPSA interaction based on proteomics analysis. A Cytoplasmic phosphorylated proteomics analysis of Eno-stimulated PBMEC for 24 h; B Nuclear proteomics analysis of Eno-stimulated PBMEC for 24 h. EIF4A2, EIF4E2, and EIF3J, the EIF family proteins, were increased in Eno-treated PBMECs (indicated as red) compared with the untreated PBMEC control (indicated as grey). [file 13567_2020_887_MOESM4_ESM.docx]

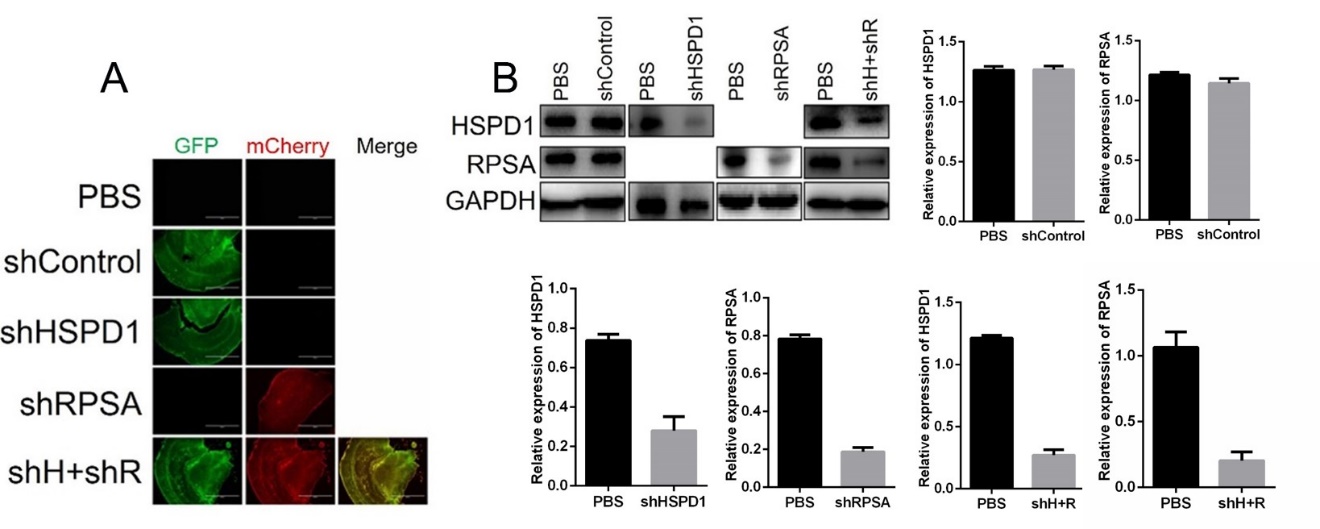

Supplement: Supplementary file 5 — Construction of the mouse model with knockdown RPSA and/or HSPD1 in the brain. A Two months after the third ventricle injection, frozen slices of the brain showed that AAV, which carried mCherry-RPSA or GFP-HSPD1, is distributed throughout the brain. Control AAV-shRNA (shControl) carried GFP (scale bar, 2000 μm); B Western blotting showed that the knockdown RPSA and/or HSPD1 model of the mouse brain was successfully established two months after the third ventricle injection by AAV, which carried mCherry-RPSA or GFP-HSPD1. [file 13567_2020_887_MOESM5_ESM.docx]

**
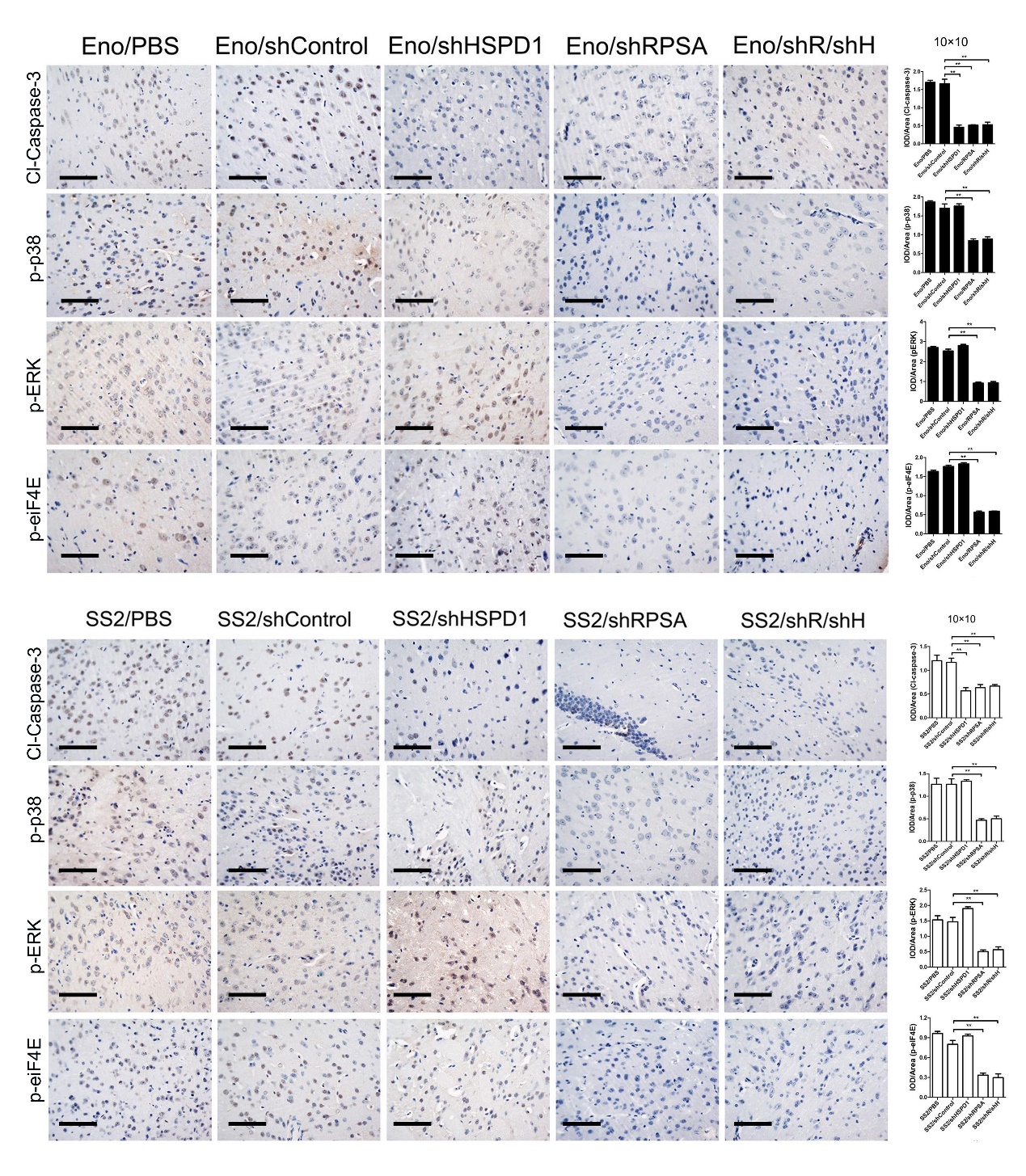
**

Supplement: Supplementary file 6 — Verification of mechanism of Eno induced apoptosis in mice. Intravenous injection of Eno or SS2 promoted the expression of apoptosis-related molecules in mouse brains by immunohistochemical analysis (*p < 0.05, **p < 0.01) (scale bar, 100 μm). [file 13567_2020_887_MOESM6_ESM.docx]
