# Supplementary material for: Streptococcus suis serotype 2 enolase interaction with host brain microvascular endothelial cells and RPSA-induced apoptosis lead to loss of BBB integrity
Source: Vet Res. 2021 Feb 22;52:30. doi: 10.1186/s13567-020-00887-6 (PMC7898445; doi:10.1186/s13567-020-00887-6)
Supplement: Supplementary file 2 — Screening and identification of Eno binding proteins in PBMECs based on LC/LC-MS and flow cytometry analysis. A, B Thirteen different proteins in protein bands between His-Eno and His tag groups were found by IP and LC/LC-MS; C pDisplay was a eukaryotic cell membrane-displayed vector. 293T cells were transfected with pDisplay::HSPD1, RPSA, JUP, TPM4, G3BP1, PTBP1, PGAM1, HH4, HSP90AB, RPS5, PHB, RPL11, and MYO1C, respectively. After 24 h, recombinant His-Eno was added to the culture dish to adhere the transfected cells. Mouse anti-His IgG was used, and flow cytometry was carried out to analyse the adhesion rate of Eno. Eno showed stronger adhesion to cells displaying RPSA and HSPD1 compared with that of the other experimental group and vector group (labeled “untreated”). We found Eno promoted HSPD1 secretion in PBMECs at 12 h by Enzyme Linked Immunosorbent Assay (ELISA). Knockdown RPSA inhibited HSPD1 secretion induced by Eno. In order to detect the effect of extracellular increased HSPD1 on PBMECs apoptosis. Experiments were designed as followed. The experiment was divided into five groups (n = 3). Remove Sup: PBMECs were treated by 0.5 uM Eno for 12 h and culture supernatant were removed. Fresh complete medium was added and cultured for another 12 h. antiE+antiH: PBMECs were treated by 0.5uM Eno for 12 h. Eno and HSPD1 antibodies was added and PBMECs were cultured for another 12 h. antiE: PBMECs were treated by 0.5 uM Eno for 12 h and Eno antibody was added and cultured for another 12 h. Enolase: PBMECs were treated by 0.5 uM Eno for 24h and analysed by flow cytometry. AntiControl: Negative isotype rabbit IgG was added into per well. All groups PBMECs were collected and analysed by flow cytometry. [file 13567_2020_887_MOESM2_ESM.docx]

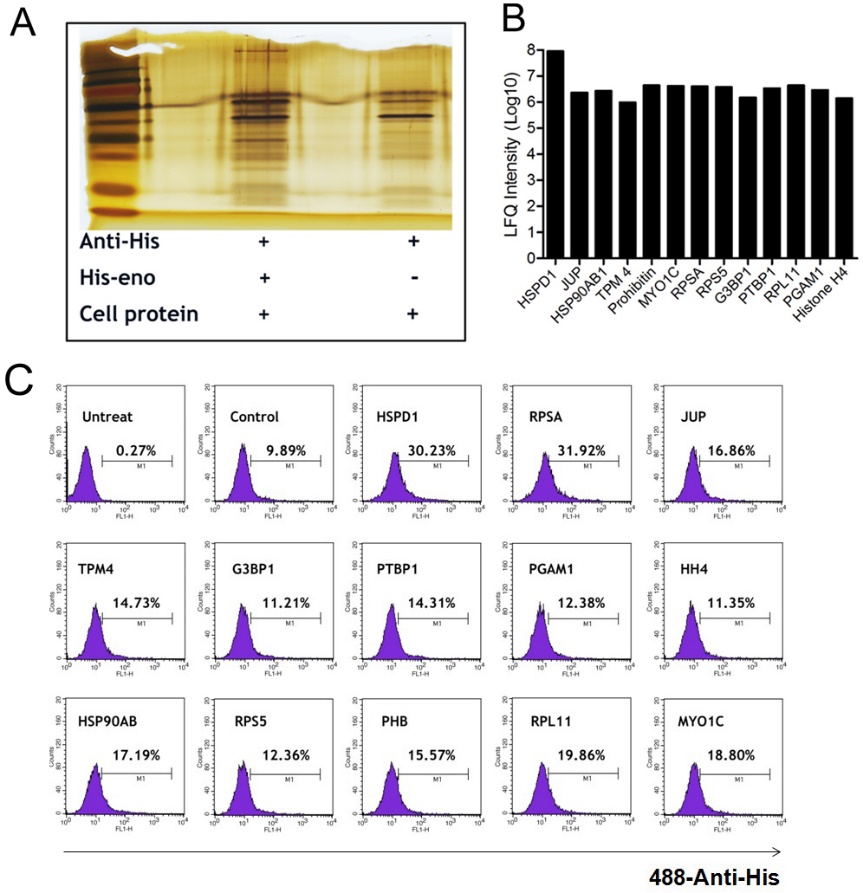


**Additional file 2 Flow cytometry analysis of Eno adhesion to 293T cells.** A, B, Thirteen different proteins in protein bands between His-Eno and His tag groups were found by IP and LC/LC-MS; C, pDisplay was a eukaryotic cell membrane-displayed vector. 293T cells were transfected with pDisplay::HSPD1, RPSA, JUP, TPM4, G3BP1, PTBP1, PGAM1, HH4, HSP90AB, RPS5, PHB, RPL11, and MYO1C, respectively. After 24 h, Recombinant His-Eno was added to the culture dish to adhere the transfected cells. Mouse anti-His IgG was used, and flow cytometry was carried out to analyse the adhesion rate of Eno. Eno showed stronger adhesion to cells displaying RPSA and HSPD1 compared with that of the other experimental group and vector group (labelled “untreated”). We found Eno promoted HSPD1 secretion in PBMECs at 12 h by Enzyme Linked Immunosorbent Assay (ELISA). Knockdown RPSA inhibited HSPD1 secretion induced by Eno. In order to detect the effect of extracellular increased HSPD1 on PBMECs apoptosis. Experiments were designed as followed. The experiment was divided into five groups (*n* = 3). **Remove Sup**: PBMECs were treated by 0.5uM Eno for 12 h and culture supernatant were removed. Fresh complete medium was added and cultured for another 12 h. **antiE+antiH**: PBMECs were treated by 0.5uM Eno for 12 h. Eno and HSPD1 antibodies was added and PBMECs were cultured for another 12 h. **antiE**: PBMECs were treated by 0.5uM Eno for 12 h and Eno antibody was added and cultured for another 12 h. **Enolase**: PBMECs were treated by 0.5uM Eno for 24h and analysed by flow cytometry. **AntiControl:** Negative isotype rabbit IgG was added into per well. All groups PBMECs were collected and analysed by flow cytometry.
