# Supplementary material for: Streptococcus suis serotype 2 enolase interaction with host brain microvascular endothelial cells and RPSA-induced apoptosis lead to loss of BBB integrity
Source: Vet Res. 2021 Feb 22;52:30. doi: 10.1186/s13567-020-00887-6 (PMC7898445; doi:10.1186/s13567-020-00887-6)
Supplement: Supplementary file 7 — 13 Proteins suspected to interact with Eno. [file 13567_2020_887_MOESM7_ESM.docx]

**Additional file 7: 13 Proteins suspected to interact with Eno**

| **Protein** | **Uniprot ID** | **Biological process** | **LFQ intensity** |
| --- | --- | --- | --- |
| HSPD1 | F1SMZ7 | [apoptotic process](http://www.ebi.ac.uk/QuickGO/GTerm?id=GO:0006919);[toll-like receptor pathway](http://www.ebi.ac.uk/QuickGO/GTerm?id=GO:0002755); [B-cell cytokine production](http://www.ebi.ac.uk/QuickGO/GTerm?id=GO:0002368); [T-cell-mediated immune response to tumour cell](http://www.ebi.ac.uk/QuickGO/GTerm?id=GO:0002842) | 89729000 |
| Jup | Q8WNW3 | [alpha-catenin binding](http://www.ebi.ac.uk/QuickGO/GTerm?id=GO:0045294); [cadherin binding](http://www.ebi.ac.uk/QuickGO/GTerm?id=GO:0045296); [signal transducer activity](http://www.ebi.ac.uk/QuickGO/GTerm?id=GO:0004871) | 2314400 |
| HSP90AB1 | F1RQU2 | [protein kinase regulator activity](http://www.ebi.ac.uk/QuickGO/GTerm?id=GO:0019887); [cellular response to interleukin-4](http://www.ebi.ac.uk/QuickGO/GTerm?id=GO:0071353); [regulation of protein ubiquitination](http://www.ebi.ac.uk/QuickGO/GTerm?id=GO:0031396); [response to stress](http://www.ebi.ac.uk/QuickGO/GTerm?id=GO:0006950) | 2699300 |
| TPM4 | D0G7F7 | [osteoblast differentiation](http://www.ebi.ac.uk/QuickGO/GTerm?id=GO:0001649) | 976920 |
| PHB | A0A0B8RZA3 | anti-proliferative protein; | 4407700 |
| MYO1C | I3LIL4 | [regulation of bicellular tight junction assembly](http://www.ebi.ac.uk/QuickGO/GTerm?id=GO:2000810); [protein targeting to membrane](http://www.ebi.ac.uk/QuickGO/GTerm?id=GO:0006612) [positive regulation of cell migration by vascular endothelial growth factor-signalling pathway](http://www.ebi.ac.uk/QuickGO/GTerm?id=GO:0038089) | 4160100 |
| RPSA | Q4GWZ2 | [Host-cell receptor for](http://www.uniprot.org/keywords/KW-1183) prion proteins, viruses, and bacteria; [Receptor](http://www.uniprot.org/keywords/KW-0675), [Ribonucleoprotein](http://www.uniprot.org/keywords/KW-0687)，[Ribosomal protein](http://www.uniprot.org/keywords/KW-0689) | 4021200 |
| RPS5 | F2Z5E6 | [Ribonucleoprotein](http://www.uniprot.org/keywords/KW-0687), [Ribosomal protein](http://www.uniprot.org/keywords/KW-0689) | 3763200 |
| G3BP1 | F1RQB6 | [negative regulation of canonical Wnt-signalling pathway](http://www.ebi.ac.uk/QuickGO/GTerm?id=GO:0090090) | 1504900 |
| PTBP1 | F1S6R7 | [nucleotide binding](http://www.ebi.ac.uk/QuickGO/GTerm?id=GO:0000166); [poly(A) RNA binding](http://www.ebi.ac.uk/QuickGO/GTerm?id=GO:0044822) | 3363100 |
| RPL11 | I3LD74 | [poly(A) RNA binding](http://www.ebi.ac.uk/QuickGO/GTerm?id=GO:0044822); [structural constituent of ribosome](http://www.ebi.ac.uk/QuickGO/GTerm?id=GO:0003735) | 4381400 |
| PGAM1 | F1S8Y5 | [Isomerase](http://www.uniprot.org/keywords/KW-0413); [Glycolysis](http://www.uniprot.org/keywords/KW-0324) | 2898300 |
| HH4 | P62802 | [DNA-binding](http://www.uniprot.org/keywords/KW-0238); Formation of the beta-catenin: TCF transactivating complex; Oxidative-stress-induced senescence | 1389900 |
