# Supplementary material for: Streptococcus suis serotype 2 enolase interaction with host brain microvascular endothelial cells and RPSA-induced apoptosis lead to loss of BBB integrity
Source: Vet Res. 2021 Feb 22;52:30. doi: 10.1186/s13567-020-00887-6 (PMC7898445; doi:10.1186/s13567-020-00887-6)
Supplement: Supplementary file 8 — siRNA used for RPSA and HSPD1 knockdown. [file 13567_2020_887_MOESM8_ESM.docx]

**Additional file 8 siRNA used for RPSA and HSPD1 knockdown**

| **Gene Name** |  | **Target Seq (5’-3’)** | **GC Content (%)** |
| --- | --- | --- | --- |
| RPSA |  | GGACCTTCACTAACCAGAT | 47.40% |
|  |  | CCACCATTGCTCTGTGTAA | 47.40% |
|  |  | GCTGTGACCAAGGAGGAAT | 52.60% |
| HSPD1 |  | GCAGATGCTGTAGCTGTTA | 47.37% |
|  |  | GCTGTAATTGCTGAACTTA | 36.84% |
|  |  | CCAGCCTTGGATTCATTAA | 42.11% |
